# The Relationship Between Job Demands and Job Stress Among Home Visiting Nurses: The Moderating Role of Job Resources

**DOI:** 10.1002/nop2.70916

**Published:** 2026-10-19

**Authors:** Jiao Xiong, Li Dai, Yu‐Jiao Sun, Li Zhang

**Affiliations:** ^1^ Guangxi University of Chinese Medicine Nanning China; ^2^ The People's Hospital of Baoan Shenzhen Shenzhen China

**Keywords:** home visiting nurses, job demands, job resources, job stress, moderating effect

## Abstract

**Aim:**

To assess the current level of job stress among home visiting nurses; to examine the correlations among job demands, job resources and job stress; and to explore whether job resources moderate the association between job demands and job stress.

**Design:**

A cross‐sectional survey.

**Methods:**

A total of 434 home visiting nurses from 13 comprehensive public hospitals and their affiliated community health centres were recruited using the convenience sampling method. The data were collected from March through June 2024. The instruments were the General Information Questionnaire, Job Demands Questionnaire, Job Resources Questionnaire and Job Stress Scale. Univariate group comparisons of job stress were performed using *t*‐tests and one‐way analysis of variance (ANOVA). Bivariate relationships among variables were assessed with Pearson or Spearman correlation coefficients, based on data distribution. To test the hypothesized moderating effect of job resources on the relationship between job demands and job stress, a hierarchical multiple regression analysis was conducted. In the first model, statistically significant demographic and work‐related variables were entered as controls. Job demands were added in the second model, followed by the four job‐resource dimensions in the third model. The final model included the interaction term between job demands and job resources to examine the moderation effect.

**Results:**

The mean item score for job stress among home visiting nurses was 1.73 ± 0.35, job demands were 3.84 ± 0.95, and job resources were 4.54 ± 0.88. Job stress differed significantly according to the number of home visits per month, weekly working hours, experience of physical threats and visiting alone. Furthermore, there was a positive correlation between job stress and job demands, whereas a negative correlation existed between job stress and job resources. Job demands negatively correlated with job resources. The interaction between job demands and job resources was significantly associated with job stress (*B* = −0.036, 95% CI [−0.058, −0.013], *p* = 0.002) and accounted for an additional 1.1% of the variance (Δ*R* ^2^ = 0.011).

**Conclusion:**

The findings indicated that the mean job stress score among home visiting nurses was relatively low compared with the possible scale range. Job resources play a moderating role in the relationship between job demands and job stress, though the buffering effect was modest. Nurse managers should mitigate job stress among home visiting nurses.

**Implications for the Profession:**

Nurse managers should strengthen social support and career development opportunities and develop supportive systems for nurses who work independently in home and community settings.

**Patient or Public Contribution:**

With their self‐report questionnaire responses, all participants helped to make this study a success.

**Reporting Method:**

The results were reported in accordance with STROBE guidelines.

## 1 Introduction

China's rapid economic development and urbanization have led to an increased demand for home and community‐based services for older adults, who often spend more of their advanced years in poor health and with disabilities (Zeng et al. 2024). Home visiting nurses play a critical role in delivering personalized care to diverse populations, such as individuals with disabilities or dementia, immobile elderly and terminally ill patients (Eltaybani et al. 2023; Murali et al. 2024). Despite their essential role, home visiting nurses face numerous occupational accidents, including traffic accidents, pet‐related accidents during service provision, harassment from patients and needle‐related accidents, which might contribute to increased job stress among home visiting nurses (Kashiwagi and Morioka 2022).

Job stress not only affects nurses' mental health but may also lead to psychological problems such as burnout, anxiety and depression (Hetzel‐Riggin et al. 2020; Xu and Yang 2021). Prolonged high‐pressure work may increase nurses' risk of cardiovascular diseases such as hyperlipidaemia, hyperglycaemia and hypertension (Chang et al. 2021). These health problems impair nurses' work ability and reduce quality of life, as well as increase turnover rates and exacerbate the nursing shortage (Baek et al. 2019; Cao et al. 2021). Therefore, the issue of job stress among home visiting nurses needs to be a high priority for managers.

## 2 Background

Research indicates that home visiting nurses often experience significant job stress. For example, a Korean study found that home visiting nurses had high levels of job stress (Kim et al. 2018). In the United States, home care workers had high job stress (Lee and Oh 2023). Studies in India also noted that 74% of home visiting nurses reported high perceived stress (Singh et al. 2021). In Turkey, nurses in home health services often exhibit negative emotions such as high stress, social isolation and increased workload (Karakaya and Uçar 2023). In China, home visiting nurses have higher job stress than hospital nurses and community centre caregivers (Yang 2009), with nearly 40% of home visiting nurses feeling significant job stress (Dai et al. 2024).

According to the Job Demands‐Resources Theory (JD‐R theory) (Bakker and Demerouti 2007), job stress is a psychological response triggered by an imbalance between job demands and job resources. Job demands refer to the physical, psychological and social challenges that individuals encounter in the workplace. These demands are often viewed as negative factors that deplete an individual's energy and resources (Bakker and Demerouti 2007). Research has shown that high job demands are positively associated with higher levels of job stress. For example, Tsen et al. (2023) found a significant positive correlation between job demands and job stress in home care services in a study of 34 European countries.

On the other hand, job resources refer to a variety of resources that can help individuals achieve their job goals, reduce the exertion caused by job demands, and promote personal growth, which may include physical, psychological, social and organizational resources (Bakker and Demerouti 2007). Research has shown that adequate job resources can alleviate the stress associated with the demands of the job and improve job performance and satisfaction (Wang et al. 2023; Yasin et al. 2019). For example, a study by Van Wingerden et al. (2018) noted that abundant job resources can significantly enhance job engagement and satisfaction. On the contrary, a lack of job resources is associated with higher job stress (Yasin et al. 2019).

The moderating role of job resources between job demands and job stress has also received a lot of attention. Ghanayem et al. (2020) found in their study of haemodialysis nurses in Israel that job resources mitigated the negative effects of high job demands on job stress. Additionally, there was evidence to support the moderating role of job resources in the association between job demands and job stress in a study by Wang and Liu (2021). While a great deal of research has been done on the relationship between job demands and job stress and the moderating effect of job resources, most studies have focused on nurses working in specialty care settings rather than home visiting nurses. Previous studies regarding job resources, job demands and job stress are limited by small sample sizes, which limits the generalizability of findings. To date, research on job stress among home visiting nurses and the moderating role of job resources in the relationship between job demands and job stress remains relatively limited. Home visiting services are largely provided by nurses from comprehensive public hospitals as part of the ‘Internet + Nursing Services’ policy. Therefore, understanding job stress in this specific hospital‐based home visiting nursing population is particularly relevant to the Chinese healthcare context. Based on the above literature, the following hypotheses were formulated in this study:

*The job demands of home visiting nurses are positively related to job stress*.

*Home visiting nurses' job resources are negatively related to job stress*.

*Job resources negatively moderated the relationship between job demands and job stress*.

## 3 The Study

### 3.1 Aims

The purposes of this study were to: (1) assess the current level of job stress among home visiting nurses; (2) examine the correlations among job demands, job resources and job stress; and (3) explore whether job resources moderate the association between job demands and job stress.

## 4 Methods

### 4.1 Design

A cross‐sectional survey design was employed. The results were reported in accordance with STROBE (Strengthening the Reporting of Observational Studies in Epidemiology) guidelines (see Data S1—STROBE checklist).

### 4.2 Setting

The study was carried out at 13 comprehensive public hospitals and their affiliated community health centres in Guangdong and Guangxi in the People's Republic of China from March through June 2024.

### 4.3 Sampling and Sample Size

With the convenience sampling method, home visiting nurses were recruited from the participating hospitals and their affiliated community health centres. The inclusion criteria were home health care nurses who (1) engaged in clinical nursing; (2) held a licence to practice as a nurse; (3) participated in services such as home visiting and Internet nursing within the past year; and (4) provided informed consent and voluntarily participated in the study. Nurses not affiliated with the participating institutions were excluded. With the cross‐sectional study sample size formula by Cao and Wu (2021), $n=\frac{\mu_{\alpha /2}^{2}\sigma^{2}}{\delta^{2}}$ and previous study by Kim et al. (2018), the standard deviation of job stress of home visiting nurses was 11.83, *α* was 0.05, the allowable error was set at 1.5, and *μ*0.05/2 = 1.96. Substituting them into the formula, the estimated sample size was 239. Considering a 20% sample failure rate, the total required sample size for the study was at least 287. To maximize recruitment across the 13 participating hospitals, all potentially eligible nurses available during the study period were invited to participate, and 511 nurses were initially screened for eligibility.

### 4.4 Data Collection

Data were collected through both online and paper‐based questionnaires using the Questionnaire Star applet. Hospitals were selected based on their established collaborative relationship with the research team and their willingness to participate. The number of home visiting nurses at each hospital was confirmed with the head nurses before the questionnaire was distributed. After obtaining head nurse approval, the questionnaire was sent to them, who then distributed it to all home visiting nurses in a standard manner via the WeChat group. To improve the response rate, a reminder was sent if the questionnaire remained unfinished after 3 days. Hospitals with < 10% online responses opted to supplement data collection via paper questionnaires to enhance participation. The same questionnaire was used for both the online and offline versions. The questionnaires were completed anonymously after informed consent was obtained, and the researcher made sure that all participants understood the purpose, content and completion requirements of the survey before distributing them. For the online format, only one submission per IP address was permitted, and all questions were required to be answered. For offline data collection, participants were verbally screened to confirm they had not previously completed the online questionnaire. Paper questionnaires were double‐entered and cross‐verified. In compliance with ethical standards, all information was kept completely confidential.

### 4.5 Measurements

The questionnaire consisted of four parts: General Information, Job Demands Questionnaire, Job Resources Questionnaire and the Community Nurse Job Stress Scale. The General Information Questionnaire was developed by the research team for this study to collect demographic and work‐related characteristics, such as age, gender, marital status, education level, professional title, job tenure, experience in home visiting nursing (years), workplace, average income, experience of physical threats, and the number of home visits and training sessions completed in the past year.

The Chinese version of the Job Demands Questionnaire (Zhang 2007) comprises 19 items across four dimensions: role conflict, work–family conflict, role ambiguity and work overload. A 7‐point Likert scale is used, ranging from 1 (strongly disagree) to 7 (strongly agree), with total scores ranging from 19 to 133; higher scores reflect greater job demands. Dimensions of work overload, role conflict and work–family conflict are scored positively, while role ambiguity (Items 6–10) is reverse‐scored. The questionnaire demonstrated a Cronbach's alpha coefficient of 0.874, with factor loadings ranging from 0.517 to 0.923, indicating sound reliability and validity. This instrument has also been shown to have acceptable reliability in a nursing sample (Wang 2023). In the present study, the Cronbach's alpha coefficient of the questionnaire was 0.903.

The Chinese version of the Job Resources Questionnaire (Zhang 2007) comprises 22 items across four dimensions: job autonomy, social support, performance feedback and career development opportunities. A 7‐point Likert scale is used, ranging from 1 (strongly disagree) to 7 (strongly agree), with total scores ranging from 22 to 154; higher scores indicate greater availability of job resources. Dimensions of job autonomy and social support are scored positively, while Item 18 under performance feedback and Items 21 and 22 under career development opportunities are reverse‐scored. The questionnaire demonstrated a Cronbach's alpha coefficient of 0.916, with factor loadings ranging between 0.575 and 0.853, indicating sound reliability and validity. This instrument has also been shown to have acceptable reliability in a nursing sample (Wang 2023). In the present study, the Cronbach's alpha coefficient of the questionnaire was 0.911.

The Community Nurse Job Stress Scale, developed by Zhu et al. (2020), comprises 25 items across four dimensions: management and interpersonal relationships, patient care, work environment and resources, and career promotion. Total scores range from 25 to 100. A 4‐point Likert scale is used (1 = never, 2 = occasionally, 3 = frequently, 4 = always), with higher scores reflecting greater stress levels. The original scale demonstrated good reliability and validity, with a Cronbach's alpha of 0.940 and factor loadings ranging from 0.507 to 0.835. Confirmatory factor analysis (CFA) yielded the following fit indices: goodness‐of‐fit index (GFI) = 0.941, adjusted goodness‐of‐fit index (AGFI) = 0.902, comparative fit index (CFI) = 0.973, root mean square error of approximation (RMSEA) = 0.045 and chi‐squared/degrees of freedom (*χ* ^2^/df) = 1.977. In the present study, the Cronbach's alpha coefficient of the scale was 0.893.

### 4.6 Statistical Analysis

The data were analysed using Statistical Package for the Social Sciences (SPSS) 26.0. Five participants each had one missing value for the variable measuring the number of home visit training sessions attended in the past year, corresponding to a missing rate of 1.15% (5/434). No missing data were observed for the study scales or any other variables. As no participants had excessive missing data, no cases were excluded. Given the very low proportion of missing data and the fact that missingness occurred only in one background variable, the five missing values were imputed using the sample mean for that variable. Mean imputation was therefore performed at the item level rather than at the scale level. This approach was considered appropriate given the minimal amount of missing data and was consistent with the recommendation of Song and Wan (2020) for datasets with a low proportion of missing data.

Normality of continuous variables was assessed using the Shapiro–Wilk test. Normally distributed continuous variables were summarized as mean ± standard deviation (SD), whereas non‐normally distributed variables were summarized as median (interquartile range). Categorical variables were presented as frequencies and percentages. Differences in job stress across demographic and work‐related characteristics were examined using independent‐samples *t*‐tests and one‐way ANOVA. When the one‐way ANOVA was statistically significant, post hoc pairwise comparisons were performed using Tukey's honestly significant difference (HSD) test. Pearson correlation coefficients were computed for normally distributed variables, and Spearman's rank correlation coefficients for non‐normally distributed variables.

Hierarchical multiple regression analysis was conducted to examine the moderating role of job resources in the association between job demands and job stress. The coding and assignment methods of variables included in the regression analysis are presented in Table S1. Regression assumptions were assessed before interpretation of the models. Linearity and homoscedasticity were examined using scatterplots of standardized residuals against standardized predicted values. Normality of residuals was assessed using histograms and normal P–P plots. Multicollinearity was evaluated using tolerance and variance inflation factor (VIF) values.

Demographic and work‐related variables showing statistically significant bivariate associations with job stress were entered as covariates in Model 1, followed by the overall job demands score in Model 2. The four job‐resource dimensions were entered simultaneously as separate predictors in Model 3 to examine their unique associations with job stress while controlling for the other dimensions. In Model 4, the interaction between overall job demands and overall job resources was entered to test H3. The overall job demands and job resources scores were mean‐centred before construction of the interaction term. The overall job resources score, rather than dimension‐specific scores, was used for the interaction because H3 concerned the overall moderating role of job resources. Predictors were entered using the enter method, and unstandardized regression coefficients (*B*) were reported. The significant interaction was plotted at 1 SD below and above the mean of job resources to illustrate the moderating pattern. Statistical significance was set at a two‐sided *p* < 0.05.

### 4.7 Ethical Considerations

The study received approval from the Ethics Committee of The Hospital (approval number: BYL20240401; approved 19 May 2024). In addition, permission was obtained from the respective authorities of study settings. Informed consent was obtained from the study subjects, and participation was voluntary.

## 5 Results

### 5.1 General Information About the Participants

Of the 511 nurses initially screened, 434 (84.9%) were eligible and included. The other 77 were excluded due to illogical responses, uniform answers or patterned responding. Missing data were handled as described in the Statistical Analysis section. 99.5% of participants were female, aged between 22 and 56 years, with an average of 4 years' experience in home care work. 54.4% of nurses worked 41 to 50 h per week, 10.6% had experienced physical threats, and 78.6% were visiting alone. For further details, please refer to Table 1.

**TABLE 1:** Job stress among home visiting nurses with different demographic characteristics (*n* = 434).

Variables	Items	*N* (%)	Mean ± SD	*t*/*F*	*p*	HSD
Gender	Male	2 (0.5)	1.84 ± 0.79	0.427^a^	0.670	—
	Female	432 (99.5)	1.73 ± 0.35			
Workplace	Tertiary hospital	99 (22.8)	1.70 ± 0.35	1.080^b^	0.340	—
	Secondary hospital	33 (7.6)	1.68 ± 0.37			
	Community health	302 (69.6)	1.75 ± 0.35			
Marital status	Unmarried	45 (10.4)	1.84 ± 0.42	1.739^b^	0.158	—
	Married	379 (87.3)	1.72 ± 0.34			
	Divorced	8 (1.8)	1.71 ± 0.30			
	Other	2 (0.5)	1.94 ± 0.25			
Education level	Secondary school	4 (0.9)	1.65 ± 0.40	0.206^b^	0.892	—
	College	61 (14.1)	1.76 ± 0.38			
	Undergraduate	362 (83.4)	1.73 ± 0.35			
	Master and above	7 (1.6)	1.70 ± 0.48			
Professional title	Nurse	17 (3.9)	1.91 ± 0.42	1.579^b^	0.179	—
	Senior nurse	63 (14.5)	1.78 ± 0.43			
	Supervisor nurse	297 (68.4)	1.72 ± 0.34			
	Associate chief nurse	54 (12.4)	1.73 ± 0.31			
	Chief nurse	3 (0.7)	1.72 ± 0.11			
Job tenure	≤ 5 years	27 (6.2)	1.83 ± 0.36	0.909^b^	0.440	—
	6–10 years	66 (15.2)	1.73 ± 0.43			
	11–15 years	69 (15.9)	1.76 ± 0.38			
	> 15 years	272 (62.7)	1.72 ± 0.32			
Number of home visit training sessions attended in the past year	≤ 1 time	149 (34.3)	1.74 ± 0.35	0.445^b^	0.641	—
	2–5 times	173 (39.9)	1.71 ± 0.37			
	> 5 times	112 (25.8)	1.75 ± 0.33			
Type of employment	Permanent employees	103 (23.7)	1.72 ± 0.32	0.332^b^	0.723	—
	Contract employees	325 (74.9)	1.74 ± 0.36			
	Dispatch system	6 (1.4)	1.77 ± 0.14			
Number of home visits in the past year (per month)	< 5 times (1)	150 (34.6)	1.66 ± 0.34	8.200^b^	< 0.001	1 < 4; 3 < 4
	5–10 times (2)	105 (24.2)	1.75 ± 0.34			
	11–30 times (3)	66 (15.2)	1.67 ± 0.30			
	> 30 times (4)	113 (26.0)	1.86 ± 0.37			
Average income (per month)	< 5000 RMB	55 (12.7)	1.75 ± 0.40	1.506^b^	0.212	—
	5000–10,000 RMB	240 (55.3)	1.76 ± 0.35			
	10,000–20,000 RMB	126 (29.0)	1.68 ± 0.33			
	> 20,000 RMB	13 (3.0)	1.73 ± 0.24			
Hours of work (per week)	≤ 40 h (1)	85 (19.6)	1.65 ± 0.37	8.163^b^	< 0.001	1 < 3; 1 < 4; 2 < 3; 2 < 4
	41–50 h (2)	236 (54.4)	1.70 ± 0.33			
	51–60 h (3)	81 (18.7)	1.83 ± 0.31			
	> 60 h (4)	32 (7.4)	1.94 ± 0.44			
Experience of physical threats	No	388 (89.4)	1.70 ± 0.33	−5.794^a^	< 0.001	—
	Yes	46 (10.6)	2.00 ± 0.40			
Visiting alone	No	93 (21.4)	1.63 ± 0.27	−3.882^a^	< 0.001	—
	Yes	341 (78.6)	1.76 ± 0.36			

*Note:* ‘a’ indicates the *t* value, ‘b’ indicates the *F* value, and ‘—’ indicates not applicable. Tukey's honestly significant difference (HSD) test was used for post hoc pairwise comparisons following a statistically significant one‐way ANOVA. For home visits per month, *F* = 8.200, *p* < 0.001: 1 < 4 (*p* < 0.001) and 3 < 4 (*p* = 0.002); all other pairwise comparisons were not statistically significant. For Hours of work (per week), *F* = 8.163, *p* < 0.001: 1 < 3 (*p* = 0.004), 1 < 4 (*p* < 0.001), 2 < 3 (*p* = 0.022) and 2 < 4 (*p* = 0.002); all other pairwise comparisons were not statistically significant. Post hoc tests were not applicable to two‐group comparisons.

### 5.2 Job Demands, Job Resources and Job Stress Scores Among Home Visiting Nurses

The mean item scores for job demands, job resources and job stress were 3.84 ± 0.95, 4.54 ± 0.88 and 1.73 ± 0.35, respectively. Among the job‐demand dimensions, work–family conflict had the highest mean score (4.64 ± 1.59). Among the job‐resource dimensions, social support had the highest mean score (5.18 ± 1.07), whereas job autonomy had the lowest (3.88 ± 1.19). Among the job‐stress dimensions, career promotion had the highest mean score (2.29 ± 0.68). Detailed scores for all dimensions are presented in Table 2.

**TABLE 2:** Job demands, job resources and job stress scores of home visiting nurses (*n* = 434).

Variables	Items	Average score
Job demands	Overall	3.84 ± 0.95
	Work overload	4.33 ± 1.38
	Role ambiguity	3.06 ± 0.95
	Role conflict	3.51 ± 1.28
	Work–family conflict	4.64 ± 1.59
Job resources	Overall	4.54 ± 0.88
	Job autonomy	3.88 ± 1.19
	Social support	5.18 ± 1.07
	Performance feedback	5.10 ± 0.90
	Career development opportunities	4.65 ± 1.17
Job stress	Overall	1.73 ± 0.35
	Management and interpersonal relationships	1.48 ± 0.41
	Patient care	1.62 ± 0.37
	Environment and resources of work	1.83 ± 0.51
	Career promotion	2.29 ± 0.68

*Note:* Values represent mean item scores. Possible score ranges are 1–7 for job demands and job resources and 1–4 for job stress.

### 5.3 Differences in Job Stress According to Demographic and Work‐Related Characteristics

Table 1 presents comparisons of job stress across demographic and work‐related characteristics. Statistically significant differences in job stress were observed according to the number of home visits per month (*F* = 8.200, *p* < 0.001), hours of work per week (*F* = 8.163, *p* < 0.001), experience of physical threats (*t* = −5.794, *p* < 0.001) and visiting alone (*t* = −3.882, *p* < 0.001).

Tukey's HSD post hoc comparisons showed that home visiting nurses who conducted > 30 home visits per month reported significantly higher job stress than those who conducted < 5 visits per month (*p* < 0.001) and those who conducted 11–30 visits per month (*p* = 0.002). No other pairwise differences in monthly home visits were statistically significant. For weekly working hours, nurses working 51–60 h per week reported significantly higher job stress than those working ≤ 40 h (*p* = 0.004) and 41–50 h (*p* = 0.022). Nurses working > 60 h per week also reported significantly higher job stress than those working ≤ 40 h (*p* < 0.001) and 41–50 h (*p* = 0.002). No other pairwise differences in weekly working hours were statistically significant.

### 5.4 The Relationship Among Job Demands, Job Resources and Job Stress of Home Visiting Nurses

The results of correlation analysis showed that job stress was positively correlated with job demands (*r* = 0.643, *p* < 0.001) and negatively correlated with job resources (*r* = −0.464, *p* < 0.001) among home visiting nurses. In addition, job demands were negatively correlated with job resources (*r* = −0.546, *p* < 0.001) (Table 3).

**TABLE 3:** Correlations among job demands, job resources and job stress of home visiting nurses (*n* = 434).

Variables	1	2	3	4	5
1. Age (years)	1				
2. Experience in home visiting nursing (years)	0.398**	1			
3. Job stress	−0.100*	0.146**	1		
4. Job demands	−0.123*	0.057	0.643***	1	
5. Job resources	0.070	−0.051	−0.464***	−0.546***	1

*Note:* Age and experience in home visiting nursing (years) were included because these demographic variables have been commonly examined as factors potentially associated with job stress in nursing research. Pearson's correlation coefficients were used for normally distributed variables, whereas Spearman's rank correlation coefficients were used for non‐normally distributed variables.

* *p* < 0.05.

** *p* < 0.01.

*** *p* < 0.001.

### 5.5 The Moderating Effect of Job Resources on the Relationship Between Job Demands and Job Stress

Hierarchical multiple regression analysis was conducted to examine the moderating effect of job resources on the relationship between job demands and job stress. There was no evidence of substantial multicollinearity, with tolerance values ranging from 0.426 to 0.877 and VIF values ranging from 1.140 to 2.349. Histograms and normal P–P plots indicated approximately normal residuals, and scatterplots of standardized residuals against standardized predicted values supported the assumptions of linearity and homoscedasticity.

As shown in Table 4, the interaction between job demands and job resources was significantly associated with job stress (*B* = −0.036, 95% CI [−0.058, −0.013], *p* = 0.002), accounting for an additional 1.1% of the variance in job stress (Δ*R* ^2^ = 0.011). Simple slope analysis was conducted at one standard deviation above and below the mean of job resources. As shown in Figure 1, the positive association between job demands and job stress was weaker at higher levels of job resources and stronger at lower levels of job resources, supporting the hypothesized buffering effect of job resources.

**TABLE 4:** Moderating effects of job resources between job demands and job stress in home visiting nurses (*n* = 434).

Variables	Model 1 (controls)			Model 2 (main effect)			Model 3 (resource dimensions)			Model 4 (interaction)		
	*B*	*p*	95% CI	*B*	*p*	95% CI	*B*	*p*	95% CI	*B*	*p*	95% CI
Constant	1.823	< 0.001	[1.609, 2.038]	0.884	< 0.001	[0.675, 1.092]	1.408	< 0.001	[1.104, 1.711]	1.377	< 0.001	[1.076, 1.678]
Age (years)	−0.008	0.004	[−0.013, −0.003]	−0.003	0.228	[−0.007, 0.002]	−0.003	0.216	[−0.007, 0.002]	−0.002	0.269	[−0.006, 0.002]
Experience in home visiting nursing (years)	0.007	0.069	[−0.001, 0.015]	0.004	0.233	[−0.002, 0.010]	0.003	0.303	[−0.003, 0.009]	0.003	0.271	[−0.003, 0.009]
Number of home visits in the past year (per month) < 5 times												
5–10 times	0.032	0.464	[−0.053, 0.116]	0.045	0.190	[−0.023, 0.113]	0.029	0.377	[−0.036, 0.095]	0.032	0.332	[−0.033, 0.097]
11–30 times	−0.041	0.412	[−0.139, 0.057]	−0.019	0.627	[−0.097, 0.059]	−0.013	0.735	[−0.088, 0.062]	−0.011	0.775	[−0.085, 0.064]
30 times	0.087	0.053	[−0.001, 0.175]	0.096	0.007	[0.026, 0.166]	0.093	0.008	[0.025, 0.162]	0.092	0.008	[0.024, 0.160]
Hours of work (per week) ≤ 40 h												
41–50 h	0.059	0.156	[−0.023, 0.141]	0.015	0.663	[−0.051, 0.080]	0.033	0.304	[−0.030, 0.097]	0.035	0.281	[−0.028, 0.097]
51–60 h	0.171	0.001	[0.070, 0.272]	0.043	0.300	[−0.039, 0.126]	0.044	0.280	[−0.036, 0.123]	0.041	0.307	[−0.038, 0.119]
60 h	0.217	0.002	[0.082, 0.352]	0.055	0.325	[−0.055, 0.165]	0.076	0.160	[−0.030, 0.183]	0.066	0.221	[−0.040, 0.172]
Experience of physical threats	0.218	< 0.001	[0.112, 0.324]	0.126	0.004	[0.041, 0.211]	0.128	0.002	[0.046, 0.210]	0.121	0.003	[0.040, 0.203]
Visiting alone	0.055	0.182	[−0.026, 0.136]	0.039	0.231	[−0.025, 0.104]	0.045	0.152	[−0.017, 0.107]	0.041	0.188	[−0.020, 0.103]
Job demands	—	—	—	0.217	< 0.001	[0.190, 0.244]	0.167	< 0.001	[0.135, 0.199]	0.178	< 0.001	[0.145, 0.210]
Job autonomy	—	—	—	—	—	—	0.000	0.972	[−0.024, 0.025]	−0.001	0.946	[−0.025, 0.023]
Social support	—	—	—	—	—	—	−0.042	0.016	[−0.075, −0.008]	−0.042	0.014	[−0.075, −0.008]
Performance feedback	—	—	—	—	—	—	0.034	0.081	[−0.004, 0.072]	0.026	0.181	[−0.012, 0.064]
Career development opportunities	—	—	—	—	—	—	−0.065	< 0.001	[−0.093, −0.038]	−0.063	< 0.001	[−0.090, −0.036]
Job demands × Job resources	—	—	—	—	—	—	—	—	—	−0.036	0.002	[−0.058, −0.013]
*F*	7.853			33.307			29.098			28.445		
Δ*F*	7.853			242.936			9.845			9.633		
*R* ^2^	0.157			0.465			0.511			0.522		
Δ*R* ^*2*^	0.157			0.308			0.046			0.011		

*Note:* The four job‐resource dimensions were entered as separate predictors in Model 3 to examine their unique contributions to job stress. The interaction term in Model 4 was constructed using the mean‐centred overall job demands and overall job resources scores. The overall job resources score, rather than dimension‐specific scores, was used for the interaction term to test the overall buffering effect hypothesized in Hypothesis 3 while limiting the number of interaction terms and potential multicollinearity. The reference categories were < 5 home visits per month and ≤ 40 h of working per week. ‘—’ indicates not applicable or a reference category, as appropriate. VIF values ranged from 1.140 to 2.349, indicating no substantial multicollinearity.

Abbreviations: Δ*F*, *F* change; Δ*R* ^2^, *R* ^2^ change; *B*, unstandardized coefficient; CI, confidence interval.

**FIGURE 1:**
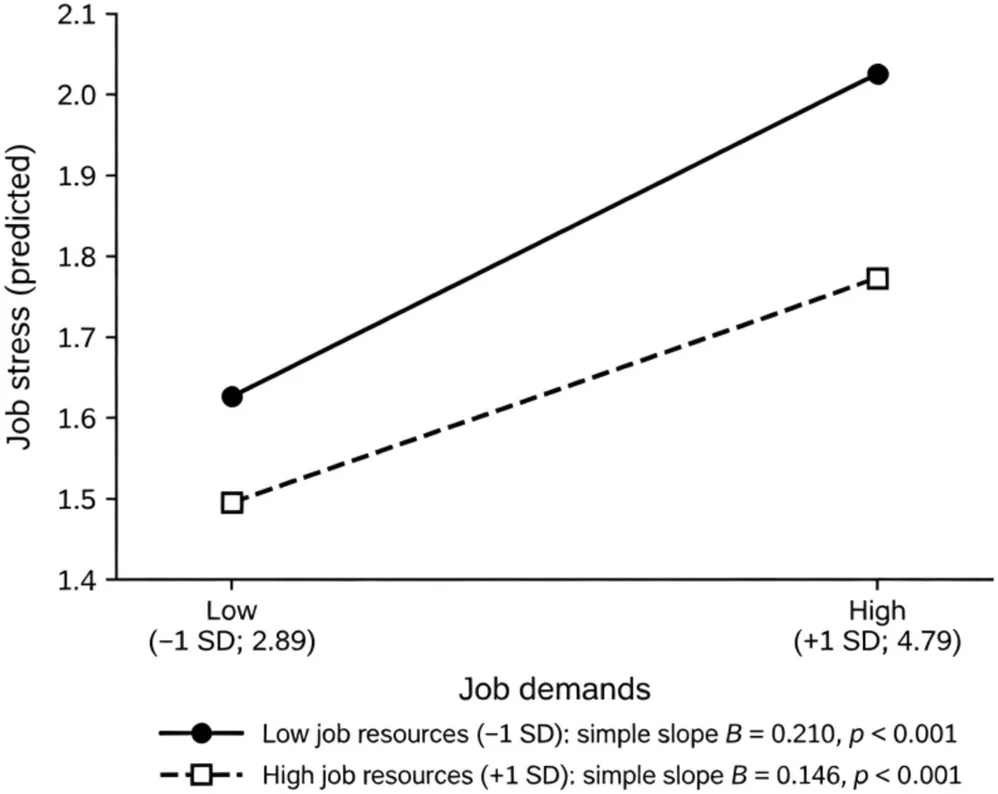
Moderating effect of job resources on the association between job demands and job stress. Predicted job stress scores were derived from Model 4 of the hierarchical multiple regression analysis (Table 4). Low and high job resources denote 1 SD below and above the sample mean for job resources, respectively. Simple slope analyses indicated that the positive association between job demands and job stress was significant at both levels of job resources but was weaker when job resources were high (*B* = 0.146, SE = 0.019, 95% CI [0.108, 0.184], *p* < 0.001) than when they were low (*B* = 0.210, SE = 0.019, 95% CI [0.172, 0.248], *p* < 0.001).

## 6 Discussion

This study examined job stress among home visiting nurses and the moderating role of job resources in the association between job demands and job stress. Several findings deserve emphasis. First, the mean job stress score was relatively low compared with the possible scale range, although career promotion had the highest mean score among the four dimensions of job stress. Second, job demands were strongly and positively correlated with job stress, whereas job resources were negatively associated with job stress. Meanwhile, job demands were negatively correlated with job resources. Finally, job resources showed a statistically significant but modest buffering effect on the association between job demands and job stress.

The results of this study showed that the job stress score of home visiting nurses was (1.73 ± 0.35), which was lower than the findings of Zhu et al. (2020) among community nurses in Sichuan, China. This may be related to differences in sample characteristics, working conditions and cultural background. Among the four dimensions of job stress, management and interpersonal relationships (1.48 ± 0.41), patient care (1.62 ± 0.37), and environment and resources of work (1.83 ± 0.51) scored lower. The results were much lower than those in the study by Guo et al. (2019), which found that management and interpersonal relationships (1.78 ± 0.59), patient care (2.22 ± 0.50), and environment and resources of work (2.49 ± 0.74). The reasons for this discrepancy may be related to differences in the subjects studied and the timing of the surveys.

Among the subscales, career promotion had the highest score (2.29 ± 0.68), indicating that home visiting nurses face greater pressure in career promotion. Studies have shown that difficulties in career promotion may lead to negative emotions such as disappointment, depression and anxiety among nurses, which can increase job stress (Ma et al. 2022). Therefore, nursing administrators should pay more attention to home visiting nurses in terms of career promotion.

Our study found that the job demands of home visiting nurses had a strong positive relationship with job stress (*r* = 0.643, *p* < 0.001). This suggests that as job demands increase, so does the level of stress experienced by nurses. These findings align with previous research indicating that high job demands, such as emotional demands and heavy workload in home care settings, contribute to elevated stress levels in home visiting nurses (Yasin et al. 2019). In addition, this may be because when nurses' job demands are too high, it often leads to difficulties in properly managing the relationship between work and family, which triggers higher levels of work–family conflict and consequently job stress (Zhang et al. 2023).

Our study also revealed that home visiting nurses' job resources were negatively correlated with job stress (*r* = −0.464, *p* < 0.001). This finding suggests that as job resources increase, the level of stress experienced by nurses decreases. These findings are consistent with the Job Demands‐Resources (JD‐R) model, which posits that while job demands contribute to stress, job resources can mitigate the negative impact of those demands (Bakker and Demerouti 2007). Our finding is also consistent with the findings of Malaysian scholars (Yasin et al. 2019). The reason may be that when nurses have sufficient job resources, it helps to promote individual mental health and alleviate the negative emotions caused by job stress, as well as moderate the sense of stress felt by the individual (Wu et al. 2024).

In addition, this study found that home visiting nurses' job demands were negatively correlated with job resources (*r* = −0.546, *p* < 0.001), which may be due to the dynamic nature of job resources, and having certain job resources such as social support, job autonomy, as well as high‐performance rewards, and career development opportunities can stimulate individuals' dedication to their jobs (Jiang et al. 2022), promote the growth and development of home visiting nurses, and reduce the fatigue associated with job demands, which in turn affects the level of job demands.

The moderation analysis showed a statistically significant interaction between overall job demands and overall job resources (*B* = −0.036, 95% CI [−0.058, −0.013], *p* = 0.002, Δ*R* ^2^ = 0.011). Specifically, the positive association between job demands and job stress was weaker at higher levels of job resources. According to Bakker and Demerouti (2007), the result of this study aligns with the Job Demands‐Resources (JD‐R) model, which posits that while job demands can lead to burnout and stress, the availability of job resources—such as social support, feedback, autonomy and development opportunities—can act as a buffer. Bakker and Demerouti (2017) mentioned that job resources can be conceptualized as an energy reservoir that supports the employee when confronted with workplace stressors. However, the small additional variance explained by the interaction term indicates that this buffering effect was modest.

At the level of specific job‐resource dimensions, hierarchical multiple regression analysis (Model 4) was adopted to examine the associations of individual job‐resource dimensions with job stress. After adjusting for covariates and other resource dimensions, career development opportunities (*B* = −0.063, 95% CI [−0.090, −0.036], *p* < 0.001) and social support (*B* = −0.042, 95% CI [−0.075, −0.008], *p* = 0.014) were independently associated with lower job stress. These findings suggest that career development opportunities and social support may be particularly relevant job resources for stress management among home visiting nurses.

As Zhang (2007) illustrated, career development opportunities reflect the extent to which the work environment provides opportunities for learning and professional development. Such opportunities may facilitate knowledge exchange, enhance professional competence and strengthen work motivation (Tsen et al. 2023). Previous research has also linked career development opportunities with greater motivation and work engagement and lower professional burnout (Kandel and Kaphle 2026; Yan and Zhang 2026). Thus, providing opportunities for continued learning and career development may be particularly relevant to stress management among home visiting nurses. In terms of social support, two separate protective mechanisms exist for home visiting nurses who work independently away from hospital teams. First, social support provides individuals with tangible practical assistance in their work, which directly reduces the operational burden brought by heavy job demands (Qin et al. 2025). Second, multi‐level support from colleagues, supervisors and administrative leaders delivers sustained emotional comfort, alleviating anxiety, exhaustion and other negative emotions triggered by high workload (Bareket‐Bojmel et al. 2021; Izadi et al. 2023).

## 7 Limitations

Several limitations of this study need to be considered. First, participants were recruited using convenience sampling from two provinces. This sampling approach may restrict the generalizability of the findings to home visiting nurses practicing in other regions or healthcare settings. In addition, convenience sampling may have introduced selection bias, and possible nonresponse bias cannot be ruled out. Second, all data were collected using self‐reported questionnaires, which may have introduced response bias. Furthermore, because both predictor and outcome variables were reported by the same respondents using the same survey method, common method bias may also have occurred. The mixed use of online and paper‐based questionnaires may have introduced mode effects and affected the consistency of data collection. Third, the cross‐sectional design precludes causal inference and does not allow determination of the temporal relationships among job demands, job resources and job stress. Fourth, hospital‐level clustering was not accounted for in the statistical analyses, which may have affected the estimation of standard errors. Finally, although the mean job stress score was relatively low compared with the possible scale range, validated cutoff scores for this instrument were not available. Therefore, the findings should not be interpreted as indicating a clinically or empirically established low‐stress category and should be interpreted with caution. Future studies should consider probability sampling across broader geographic regions, longitudinal or prospective designs, multi‐source data collection, standardized survey administration and multilevel modelling to address these limitations.

## 8 Conclusion

The findings revealed that overall, the mean job stress score among home visiting nurses was relatively low compared with the possible scale range. A positive association between job demands and stress levels was discovered, suggesting that as job demands rise, so does the stress level that nurses encounter. Conversely, job stress was negatively correlated with job resources, suggesting that access to resources such as social support and career development opportunities helps to alleviate stress.

The study also highlighted that job resources play a moderating role in the relationship between job demands and job stress, though the buffering effect was modest. The major stakeholders should design a well‐informed intervention aimed at increasing job resources, particularly social support and opportunities for career development. By fostering a supportive work environment and offering avenues for professional growth, organizations can help nurses better manage job demands and maintain their well‐being.

## 9 Implications for Nursing

For nursing practice, nurse managers should prioritize enhancing job resources, particularly social support and career development opportunities, to mitigate stress. For nursing leadership, structured support systems such as peer mentoring and regular debriefing sessions should be established to address the unique challenges of independent community‐based work. For nursing education and policy, career advancement pathways and resource allocation strategies should be integrated into community nursing workforce policies. These measures may improve nurse retention and quality of home care delivery.

## Author Contributions

**Jiao Xiong:** investigation, writing – original draft, formal analysis, writing – review and editing, methodology, conceptualization, data curation. **Li Dai:** supervision, writing – review and editing, conceptualization, methodology, writing – original draft. **Yu‐Jiao Sun:** formal analysis, writing – review and editing. **Li Zhang:** formal analysis, writing – review and editing.

## Funding

The study was funded by the Shenzhen Baoan District Medical Association, ‘Research on the Influencing Factors of Home Visiting Nurses' Job Stress Based on the Job Demands‐Resources Theory: Structural Equation Modeling Analysis’ (BAYXH2024004), and by the Shenzhen Science and Technology Program (JCYJ20210324110809027), ‘Identification of Risk Factors and Construction of a Prevention and Control System for Nurses in “Internet + Nursing Service” Based on the Reason Model’.

## Ethics Statement

The study received approval from the Ethics Committee of The Hospital (Approval Number: BYL20240401; approved 19 May 2024). In addition, permission was obtained from the respective authorities of study settings.

## Consent

Informed consent was obtained from the study subjects, and participation was voluntary.

## Conflicts of Interest

The authors declare no conflicts of interest.

## Supporting information


**Table S1:** Coding and assignment methods of variables included in the regression analysis (*n* = 434).

**Data S1:** STROBE statement—Checklist of items that should be included in reports of cross‐sectional studies.

## Data Availability

The data that support the findings of this study are available from the corresponding author upon reasonable request.
