# Supplementary material for: The Relationship Between Job Demands and Job Stress Among Home Visiting Nurses: The Moderating Role of Job Resources
Source: Nurs Open. 2026 Oct 19;13(10):e70916. doi: 10.1002/nop2.70916 (PMC13680685; doi:10.1002/nop2.70916)
Supplement: Supplementary file 1 [file NOP2-13-e70916-s001.docx]

Supplementary Table S1. Coding and assignment methods of variables included in the regression analysis (*n*=434)

| **Variables** | **Assignment method** |
| --- | --- |
| Age (years) | Substitute the original value |
| Experience in home visiting nursing (years) | Substitute the original value |
| Number of home visits in the past year (per month) | Dummy variables were set for “< 5 times” as the reference category, “5−10 times” (1, 0, 0), “11−30 times” (0, 1, 0), and “> 30 times” (0, 0, 1) |
| Hours of work (per week) | Dummy variables were set for “≤ 40 hours” as the reference category, “41−50 hours” (1, 0, 0), “51−60 hours” (0, 1, 0), and “> 60 hours” (0, 0, 1) |
| Experience of physical threats | No = 0, Yes = 1 |
| Visiting alone | No = 0, Yes = 1 |
| Job demands | Substitute the original value |
| Job resources | Substitute the original value |
| Job stress | Substitute the original value |
