# Supplementary material for: The Relationship Between Job Demands and Job Stress Among Home Visiting Nurses: The Moderating Role of Job Resources
Source: Nurs Open. 2026 Oct 19;13(10):e70916. doi: 10.1002/nop2.70916 (PMC13680685; doi:10.1002/nop2.70916)
Supplement: Supplementary file 2 [file NOP2-13-e70916-s002.docx]

STROBE Statement—Checklist of items that should be included in reports of ***cross-sectional studies***

|  | Item No | Recommendation | Page No |
| --- | --- | --- | --- |
| Title and abstract | 1 | (a) Indicate the study’s design with a commonly used term in the title or the abstract   In the abstract and methods. | Page 1  Page 5-6 |
|  |  | (b) Provide in the abstract an informative and balanced summary of what was done and what was found   Provided in abstract. | Page 1-2 |
| Introduction | | | |
| Background/rationale | 2 | Explain the scientific background and rationale for the investigation being reported  Included in the introduction and background. | Page 3-5 |
| Objectives | 3 | State specific objectives, including any prespecified hypotheses  Included in the background and aims. | Page 5 |
| Methods | | | |
| Study design | 4 | Present key elements of study design early in the paper  Included in the abstract, study design under the methods section. | Page 1 Page 5-10 |
| Setting | 5 | Describe the setting, locations, and relevant dates, including periods of recruitment, exposure, follow-up, and data collection  Included in the setting, sampling and sample size under the methods section. | Page 6-7 |
| Participants | 6 | (a) Give the eligibility criteria, and the sources and methods of selection of participants   Included in the sampling and sample size under the methods section. | Page 6 |
| Variables | 7 | Clearly define all outcomes, exposures, predictors, potential confounders, and effect modifiers. Give diagnostic criteria, if applicable  Included in the study design, sampling and sample size under the Methods section. | Page 6 |
| Data sources/ measurement | 8* | For each variable of interest, give sources of data and details of methods of assessment (measurement). Describe comparability of assessment methods if there is more than one group  Included in the data collection and measurements | Page 6-9 |
| Bias | 9 | Describe any efforts to address potential sources of bias  Described in study limitations. | Page 16- 17 |
| Study size | 10 | Explain how the study size was arrived at  Stated in the sampling and sample size, and result section. | Page 6  Page 11 |
| Quantitative variables | 11 | Explain how quantitative variables were handled in the analyses. If applicable, describe which groupings were chosen and why  Included in the statistical analysis | Page 9 Page10 |
| Statistical methods | 12 | (a) Describe all statistical methods, including those used to control for confounding   Included in the statistical analysis. | Page 9 Page10 |
|  |  | (b) Describe any methods used to examine subgroups and interactions   Included in the statistical analysis. | Page 9  Page10 |
|  |  | (c) Explain how missing data were addressed   Included in the statistical analysis. | Page9 |
|  |  | (*d*) If applicable, describe analytical methods taking account of sampling strategy | Not applicable |
|  |  | (*e*) Describe any sensitivity analyses | Not applicable |
| Results | | | |
| Participants | 13* | (a) Report numbers of individuals at each stage of study—eg numbers potentially eligible, examined for eligibility, confirmed eligible, included in the study, completing follow-up, and analysed   Included in results. | Page 11 |
|  |  | (b) Give reasons for non-participation at each stage   Included in results. | Page 11 |
|  |  | (c) Consider use of a flow diagram | Not applicable |
| Descriptive data | 14* | (a) Give characteristics of study participants (eg demographic, clinical, social) and information on exposures and potential confounders   Included in results. | Page 11  Table 1 |
|  |  | (b) Indicate number of participants with missing data for each variable of interest   Included in the statistical analysis and results. | Page 9 Page 11 |
| Outcome data | 15* | Report numbers of outcome events or summary measures  Included in results. | Page 11- 13 |
| Main results | 16 | (a) Give unadjusted estimates and, if applicable, confounder-adjusted estimates and their precision (eg, 95% confidence interval). Make clear which confounders were adjusted for and why they were included   Included in the statistical analysis、results and Table. | Page 10  Page 12  Table 4 |
|  |  | (c) Report category boundaries when continuous variables were categorized   Included in the results and Table. | Page 11  Table S1 |
|  |  | (*c*) If relevant, consider translating estimates of relative risk into absolute risk for a meaningful time period | Not applicable |
| Other analyses | 17 | Report other analyses done—eg analyses of subgroups and interactions, and sensitivity analyses | Not applicable |
| Discussion | | | |
| Key results | 18 | Summarise key results with reference to study objectives  Included in the discussion. | Page 13- 16 |
| Limitations | 19 | Discuss limitations of the study, taking into account sources of potential bias or imprecision. Discuss both direction and magnitude of any potential bias  Described in study limitations and conclusion | Page 16- 17 |
| Interpretation | 20 | Give a cautious overall interpretation of results considering objectives, limitations, multiplicity of analyses, results from similar studies, and other relevant evidence  Described in discussion. | Page 13- 16 |
| Generalisability | 21 | Discuss the generalisability (external validity) of the study results  Discussed in study limitations and conclusion. | Page 16-17 |
| Other information | | | |
| Funding | 22 | Give the source of funding and the role of the funders for the present study and, if applicable, for the original study on which the present article is based | Title page |

*Give information separately for exposed and unexposed groups.

**Note:** An Explanation and Elaboration article discusses each checklist item and gives methodological background and published examples of transparent reporting. The STROBE checklist is best used in conjunction with this article (freely available on the Web sites of PLoS Medicine at http://www.plosmedicine.org/, Annals of Internal Medicine at http://www.annals.org/, and Epidemiology at http://www.epidem.com/). Information on the STROBE Initiative is available at www.strobe-statement.org.
